# Cross-sectional study on the relationship between COVID vaccination and willingness to receive the influenza vaccine in Jeddah

**DOI:** 10.25122/jml-2025-0165

**Published:** 2025-11

**Authors:** Khaled Abdulraouf Yaghmour, Mohammed Abdu Noorh, Ali Omar Aqeeli, Faisal Khaled Ameen, Eyad Abdulmuti Dahlawi, Abdulmohsen Hilmi Sindi, Abdulaziz Turki Alhadrami, Omar Tareq Khawaji, Baraa Abdallah Alqethami, Mohammed Talal Kheyami

**Affiliations:** 1 Department of Family Medicine, King Abdulaziz University Hospital, Jeddah, Kingdom of Saudi Arabia; 2 College of Medicine, King Abdulaziz University, Jeddah, Kingdom of Saudi Arabia

**Keywords:** COVID-19 vaccination, influenza vaccine, vaccine hesitancy, vaccine acceptance, cross-sectional study, willingness to vaccinate, public perception, immunization behavior, Saudi Arabia, Jeddah

## Abstract

COVID-19 and influenza pose serious health risks, and vaccine hesitancy is a major global health challenge. This study examined how the COVID-19 pandemic influenced public awareness and willingness to receive the flu vaccine in Jeddah, the Kingdom of Saudi Arabia (KSA). A cross-sectional web-based survey was conducted in Jeddah from October 2023 to May 2024. The survey targeted individuals aged 18 or older residing in Jeddah. Of the 487 responses, 400 met the inclusion criteria. The survey, administered in Arabic, included 20 questions on demographics, awareness, and attitudes toward vaccines. Among the participants, 72.3% were men and 54.5% were aged 18–24 years. Furthermore, 67% reported increased vaccination awareness after post-COVID-19, and 48.25% did not experience increased hesitation. Nearly all participants (94%) had heard of the influenza vaccine; however, 51.5% were unaware of its role in reducing hospitalization. Only 64% reported they would have been more likely to receive the vaccine if they had been aware of this benefit. Trust in healthcare providers’ recommendations was low. Significant associations are observed between age and awareness of the influenza vaccine, and between age and perceived adequacy of vaccine information. Healthcare workers differ significantly from non-healthcare workers in their vaccine-related beliefs. The COVID-19 pandemic increased awareness of vaccines, particularly for influenza. However, hesitancy persists, driven by concerns about vaccine content and distrust toward health recommendations. Addressing these concerns is crucial for improving vaccine uptake in KSA.

## Introduction

In the 21^st^ century, the development of safe and effective vaccines has become the cornerstone of global health. In addition to providing clean water and sanitation, vaccines play a vital role in reducing global mortality from preventable diseases [1]. Smallpox vaccination programs have proven this, with 100 million dollars spent preventing 1.3 billion deaths worldwide each year [2].

On March 11, 2020, the World Health Organization (WHO) declared coronavirus disease 2019 (COVID-19) a global pandemic [3]. In contrast, influenza viruses can cause epidemics or even pandemics, unlike those caused by coronaviruses [4], affecting an estimated 5 million individuals annually and causing approximately 650,000 deaths [5]. Both infections have similar clinical presentations that affect the respiratory system and produce comparable symptoms, such as productive and non-productive cough, fever, malaise, dyspnea, and anorexia [6,7]. Evidence suggests that pandemics can influence public willingness to accept vaccines, either positively or negatively [8].

Vaccine hesitancy is defined by the WHO as a ‘delay in the acceptance or refusal of vaccines despite the availability of vaccination services’ [9]. It was one of the top 10 global threats in 2019 [10]. According to Leask, the people targeted by vaccination programs can be categorized into five groups: ‘unquestioning acceptors, cautious acceptors, hesitant parents, late or selective acceptors, and rejectors’ [11].

In Saudi Arabia, one study reported that approximately 90% of participants were vaccinated against COVID-19, while only 20% reported following international recommendations for annual influenza vaccination; however, 56% believed that COVID-19 vaccination increased their awareness of influenza vaccination [12]. A study conducted in Turkey found that 78.98% of participants were vaccinated against COVID-19; among vaccinated individuals, 55% reported self-protection and protection of others as their main motivations, while 68.7% of unvaccinated participants cited distrust in vaccine content or country of manufacture as their primary concern [13]. In Hong Kong, a study of healthcare workers found that the influenza vaccination acceptance rate was similar before the pandemic (2019) and during the pandemic (2020) [6]. A few studies have examined the impact of the COVID-19 vaccine on people’s willingness and awareness of the influenza vaccine, especially in the KSA. Therefore, this study aims to examine how COVID-19 vaccination influenced public awareness and willingness to receive the influenza vaccine in Jeddah, KSA.

## Material and Methods

### Study design

A cross-sectional, web-based survey was conducted in Jeddah, Kingdom of Saudi Arabia (KSA). The survey was distributed through social media platforms, including WhatsApp, Snapchat, X (formerly Twitter), and Telegram, between October 2023 and May 2024. An Arabic questionnaire was administered using Google Forms. The survey required approximately 2–3 minutes to complete and consisted of 20 questions covering demographic characteristics, awareness, beliefs, and attitudes toward vaccination. A validated questionnaire adapted from a study by Nada Alsuhebany [14] was used, and informed consent was obtained from all participants.

### Study population

The target population included individuals aged 18 years who resided in Jeddah. Anyone living outside of Jeddah was excluded. A total of 487 participants responded to the questionnaire, 400 of whom 400 met the inclusion criteria.

### Sampling

The sample size was calculated using the Raosoft sample size calculator for the Jeddah population, with a 95% confidence level and a 5% margin of error. The minimum required sample size was 385 participants. This study aimed to include at least 400 respondents.

### Data collection

Data were collected using a self-administered online questionnaire through Google Forms. The survey included 20 questions assessing demographic characteristics and participants’ awareness, beliefs, and attitudes toward vaccines.

### Statistical analysis

Respondents’ demographic characteristics were summarized using frequencies and percentages. Chi-square tests were used to assess differences in awareness and attitudes across demographic groups. Statistical significance was set at *P* < 0.05. All analyses were performed using SPSS version 21.

## Results

### Demographics

A total of 400 participants were included in the study. Of these, 289 (72.3%) were male. The largest age group was 18–24 years, comprising 218 participants (54.5%). Most participants held a high school diploma (177, 44.1%), and the majority were Saudi nationals (371, 92.8%). Most respondents were married (285, 71.3%), and 57 (14.3%) worked in healthcare (Table 1).

**Table 1:** Demographic characteristics of study participants

		*n*	%
Age	18–24	218	54.5%
	25–34	103	25.8%
	35–44	42	10.5%
	45–54	18	4.5%
	55–64	16	4.0%
	>65	3	0.8%
Sex	Female	111	27.8%
	Male	289	72.3%
Marital status	Married	285	71.3%
	Single	107	26.8%
	Divorced	6	1.5%
	Widow	2	0.5%
Nationality	Saudi	371	92.8%
	Non-Saudi	29	7.3%
Education	Higher education	150	37.4%
	High school diploma	177	44.1%
	Diploma	25	6.2%
	Bachler	37	9.2%
	Illiterate	4	1.0%
	Intermediate	8	2.0%
Chronic disease	Do not know	55	13.8%
	No	320	80.0%
	Yes	25	6.3%
Smoking status	Ex-smoker	57	14.3%
	No	323	80.8%
	Yes	20	5.0%
Healthcare workers	Yes	57	14.25%
	No	343	85.75%

**Figure 1:**
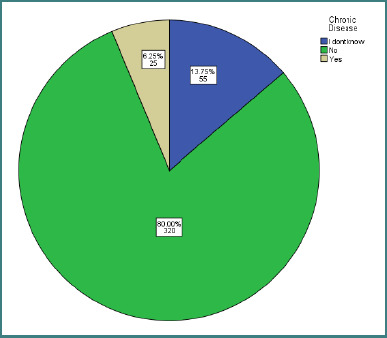
Pie chart of chronic illness distribution in the sample

A total of 320 participants (80.0%) reported no chronic disease, while 25 (6.3%) reported a chronic illness and 55 (13.8%) were unsure of their status (Figure 1).

### Awareness and attitudes toward influenza vaccination

Most participants (*n* = 376, 94%) heard about the influenza vaccine. Of these, 353 (93.9%) were Saudi citizens. Approximately 51.5% (*n* = 206) did not know that receiving the influenza vaccine could reduce the risk of hospital and intensive care unit (ICU) admissions; 191 of these (92.7%) were Saudi nationals. No statistically significant association was found between demographic variables and awareness of the vaccine’s role in preventing hospitalization and ICU admission (χ^2^ = 8.251, *P* = 0.604). This indicates that knowledge of the vaccine’s preventive benefits was not strongly influenced by age or nationality. In total, 64% (*n* = 256) stated that if they knew that the influenza vaccine could prevent hospitalization and ICU admission, they would have taken it; most of these (239, 93.4%) were Saudi nationals.

Regarding general vaccine beliefs, 225 participants (56.3%) believed that vaccines provide immunity against viral and bacterial infections; 208 (92.4%) of these were Saudi nationals. In addition, 249 participants (62.3%) believed that sufficient information about vaccine safety and efficacy is available. The belief that sufficient information is available was significantly associated with age (*P* < 0.001), but not with nationality (*P* = 0.864). Only 185 participants (46.3%) reported trusting healthcare practitioners’ vaccination recommendations. Trust in healthcare providers was not significantly associated with age (*P* = 0.776) or nationality (*P* = 0.603).

A significant association was found between healthcare worker status and willingness to receive the vaccine if it prevents hospitalization (*P* = 0.035), as well as between healthcare worker status and belief that sufficient vaccine safety information is available (*P* = 0.022).

### Changes in vaccination attitudes after COVID-19

Approximately 67% (*n* = 268) reported that the COVID-19 pandemic increased their overall awareness of vaccination. Of this group, 247 (92.16%) were Saudi citizens. In contrast, 48.25% (*n* = 193) said that the pandemic did not make them hesitant to receive other vaccines.

The chi-square test indicated that increased awareness was significantly associated with age (*P* = 0.002) and healthcare worker status (*P* = 0.036) but not with nationality (*P* = 0.666). The effect of the pandemic on vaccination hesitance was not statistically significant according to age (*P* = 0.156) or nationality (*P* = 0.274).

## Discussion

The COVID-19 vaccination has led to significant changes in public health, particularly in how people perceive and respond to vaccines. This study sought to understand how experiences with the COVID-19 vaccine influence public willingness to receive the seasonal influenza vaccine. This is one of the few studies to examine how the experience with COVID-19 vaccines shapes attitudes toward other vaccines, especially influenza vaccines.

Our findings revealed that 94% of participants were aware of the influenza vaccine, indicating strong community-wide awareness. However, only 51.5% of the participants were informed that the vaccine could lower the risk of hospital and ICU admissions, suggesting a significant gap in understanding its specific benefits. Similar results were previously reported. For instance, a study conducted in Riyadh by Tharkar [15] found low influenza vaccine uptake during the pandemic, with many participants declining vaccination due to insufficient information or concerns about potential side effects. These findings suggest that while vaccine awareness is high, understanding its effectiveness in preventing severe outcomes remains incomplete.

In our study, 64% of participants reported they would have been more likely to receive the flu vaccine if they had known it could help prevent hospitalization. This underscores the importance of targeted educational campaigns to highlight the benefits of vaccines. A national study of healthcare professionals in Saudi Arabia by Alshammari [16] revealed similar gaps in understanding, with vaccine acceptance strongly linked to awareness of health advantages. Efforts to improve vaccine uptake should focus on closing the information gaps in Saudi Arabia and elsewhere.

The pandemic has affected public attitudes toward vaccination. While 67% of the participants reported an overall increase in awareness since the pandemic, 48.25% indicated that it did not make them hesitant to receive other vaccines. This hesitancy, which may be related to concerns regarding vaccine safety and effectiveness, has been reported in other studies. A study in Turkey by Özdinç [13] found that the availability of accurate, detailed information significantly influenced vaccination decisions. Therefore, improving education and building trust are key to overcoming vaccine hesitancy, consistent with the results of Maisonneuve *et al*. [17].

A noteworthy finding in our study was that only 46.3% of participants fully trusted their healthcare providers’ recommendations regarding vaccines. This lack of trust is significant because healthcare professionals play a crucial role in influencing vaccine uptake. Studies by Alshammari [16] and Alsuhebany [14] reported that healthcare workers’ attitudes toward vaccines can significantly affect patients' acceptance of vaccines. Therefore, improving communication between healthcare providers and patients, emphasizing trust, and delivering clear, evidence-based information on vaccine safety and efficacy should be prioritized.

Demographic factors influence vaccination attitudes in our study. Most participants were male (72.3%), and a large proportion were young (54.5% aged 18–24 years). Previous studies by Tharkar [15] and Alsuhebany [14] have shown that younger individuals, particularly those with higher levels of education, have more positive attitudes toward vaccines. However, our results were mixed: 62.25% believed sufficient information was available to the public regarding vaccine safety and effectiveness, yet approximately half of the sample felt hesitant or distrustful toward healthcare recommendations. This indicates that public health campaigns must be more comprehensive, especially for younger audiences, who may rely on digital platforms for information.

While awareness of the flu vaccine is high, notable gaps in understanding its full benefits, especially regarding serious complications, persist. The COVID-19 pandemic has had a mixed impact on vaccination attitudes, with some individuals becoming more aware while others remain hesitant. Public health efforts should aim to address these knowledge gaps and rebuild trust in healthcare providers to encourage informed decision-making and boost vaccine uptake [18].

### Study limitations

This cross-sectional observational study has several limitations. It was conducted in Jeddah City; a larger study encompassing the entire KSA with a larger sample size would better represent the population. Additionally, the proportion of non-Saudi participants was notably lower than that of the population of the KSA. Furthermore, smartphone use is less prevalent among older adults at higher risk; therefore, the results may not have fully captured attitudes in this subgroup.

## Conclusion

This study showed that awareness of influenza vaccination significantly increased among individuals aged 18–24 years after the COVID-19 pandemic. Most participants believed that sufficient knowledge existed regarding vaccine safety and efficacy, and they recognized that vaccines could enhance immunity against viruses and bacteria. However, hesitation toward the influenza vaccine has not been markedly affected by the COVID-19 pandemic. We observed significant differences in awareness among age groups and between healthcare worker statuses, highlighting the need for targeted awareness strategies. Future efforts should aim to spread awareness through social media, campaigns, and educational materials in community settings.
